# Enhanced Avulsion Technique for Brachioplasty and Cruroplasty: Minimizing Complications and Ensuring Patient Safety

**DOI:** 10.7759/cureus.45390

**Published:** 2023-09-17

**Authors:** Elias Sabbagh, Nathan Ferreira, Jean Philippe Giot

**Affiliations:** 1 Plastic and Reconstructive Surgery, Centre Hospitalier Universitaire (CHU) Grenoble Alpes, Grenoble, FRA

**Keywords:** seroma, liposuction, avulsion, brachioplasty, cruroplasty

## Abstract

Background

Brachioplasty and cruroplasty are commonly performed aesthetic procedures, but they are not without their risks. Among the potential complications, the development of seroma or hematoma is particularly concerning. In this article, we present a modified avulsion technique designed to reduce complications and improve patient outcomes.

Methods

Our study included all consecutive patients (n=28) who underwent brachioplasty and/or cruroplasty using the modified avulsion technique at the Plastic Surgery Department of the University Hospital of Grenoble between September 2019 and November 2022. Data collection was conducted retrospectively to evaluate the complications of the procedure. Histological analysis was performed on samples of excised tissues from five patients operated on with the avulsion technique and five patients operated on with electrocautery resection.

Results

A total of 28 patients were reviewed, with a mean follow-up of 22 months. Among the 28 patients, regarding the Common Terminology Criteria for Adverse Events (CTCAE), there were no major complications, with minor complications occurring in 55% of the cases.

Conclusion

Dermolipectomies of the extremities are associated with a high level of patient satisfaction with a low risk of major complications. The avulsion technique practiced by the authors proved to be a safe and efficient procedure.

## Introduction

With the recent increase in bariatric surgeries, arm and thigh contouring procedures have gained increased popularity. Since the first description of brachioplasty by Posse in 1943 [1] and cruroplasty by Lewis in 1957 [2], techniques have continuously evolved, leading to lower complication rates. The adoption of novel approaches has successfully reduced the occurrence of cutaneous necrosis. The implementation of Lockwood's technique, utilizing fixation to the Colles’ fascia [3] in cruroplasty, has contributed to a reduction in scar migration. Additionally, the introduction of pre-excision liposuction by Lelouarn and Pascal [4] has effectively decreased postoperative seroma rates. The primary objective of this preliminary liposuction is to create a superficial dissection plane within the superficialis fascia, where the lymphatic vessels are located [5]. Therefore, aggressive liposuction of the resection area preserves the superficial lymphatic network, blood vessels, and nerves during the skin excision [6]. This approach consequently reduces the risk of postoperative seromas, neuromas, and hematomas [7]. Furthermore, following liposuction, the skin can be incised and avulsed from proximal to distal, minimizing the risk of vessel trauma as evoked by Hunstad and Knotts [8,9]. Severe complication rate associated with these procedures is low. However, the minor complication rate remains relatively high. These complications primarily consist of minor issues, such as small scar dehiscence related to inflammatory reactions caused by absorbable sutures.

## Materials and methods

A monocentric retrospective review was conducted on all consecutive brachioplasty or cruroplasty patients treated at the Plastic Surgery Department of Grenoble Hospital (France) between September 2019 and November 2022. Data collected included patient age, body mass index (BMI), length of follow-up, concurrent surgeries, and occurrences of complications and adverse events as described by Morzycki [10]. All procedures were performed by two highly qualified surgeons with more than 10 years of experience in aesthetic surgery. All authors have performed this procedure on patients included in the data collection for this article, which conforms to the ethical principles set forth in the Declaration of Helsinki for research on human subjects.

Surgical technique of the modified avulsion

Drawings

Cruroplasty: Scars can be positioned vertically along the axis of the extremity or horizontally in the groin crease [3] or with a combination of vertical and horizontal incisions [11]. The markings begin by drawing a line connecting the pubic symphysis to the medial condyle of the femur. This line corresponds to the ideal scar placement. It can be extended distally below the knee if needed. The width of the excision flap is estimated using the pinch test, and the markings are made with a dermographic pen.

Brachioplasty: There are different options for scar placement. The medial scar is positioned along the bicipital groove or posteriorly in line with the triceps muscle. More recently, a posteromedial incision has been described [12], located between the two previous options. None of these options provide any benefits in terms of scar quality or dehiscence compared to the others [13]. The incision line begins from the medial epicondyle and extends to the midaxillary line. If the excess skin and fat are limited to the arm, the incision stops at the axillary crease. If it extends to the lateral part of the chest, the incision is extended to the chest wall along the midaxillary line [14]. The scar is drawn with a broken line crossing with the axillary crease to prevent shoulder movement restriction. Similar to cruroplasty, the width of the resection flap is determined by the pinch test, and the markings are made with a dermographic pen. Once the patient is under anesthesia, all markings are rechecked before infiltration.

Liposuction

**Figure 1:**
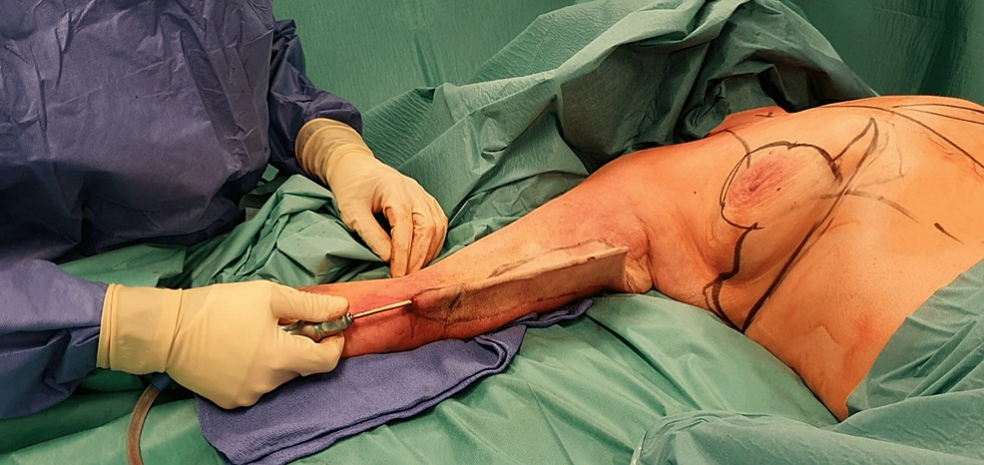
Aggressive liposuction of the entire resection area

After adequate draping, infiltration with tumescent “wet technique” epinephrine-containing saline solution (1mg/L) is performed using an atraumatic cannula. Entry points of the cannula are located in the area of skin resection. If necessary, classic liposuction can be performed outside the resection area. Following infiltration, aggressive liposuction (below the superficial fascial system, above and under the skin) is performed below the resection area. At the end of liposuction, a noticeable difference in subcutaneous fat thickness can be observed between the excision area and the scar borders (Figure 1).

Avulsion

**Figure 2:**
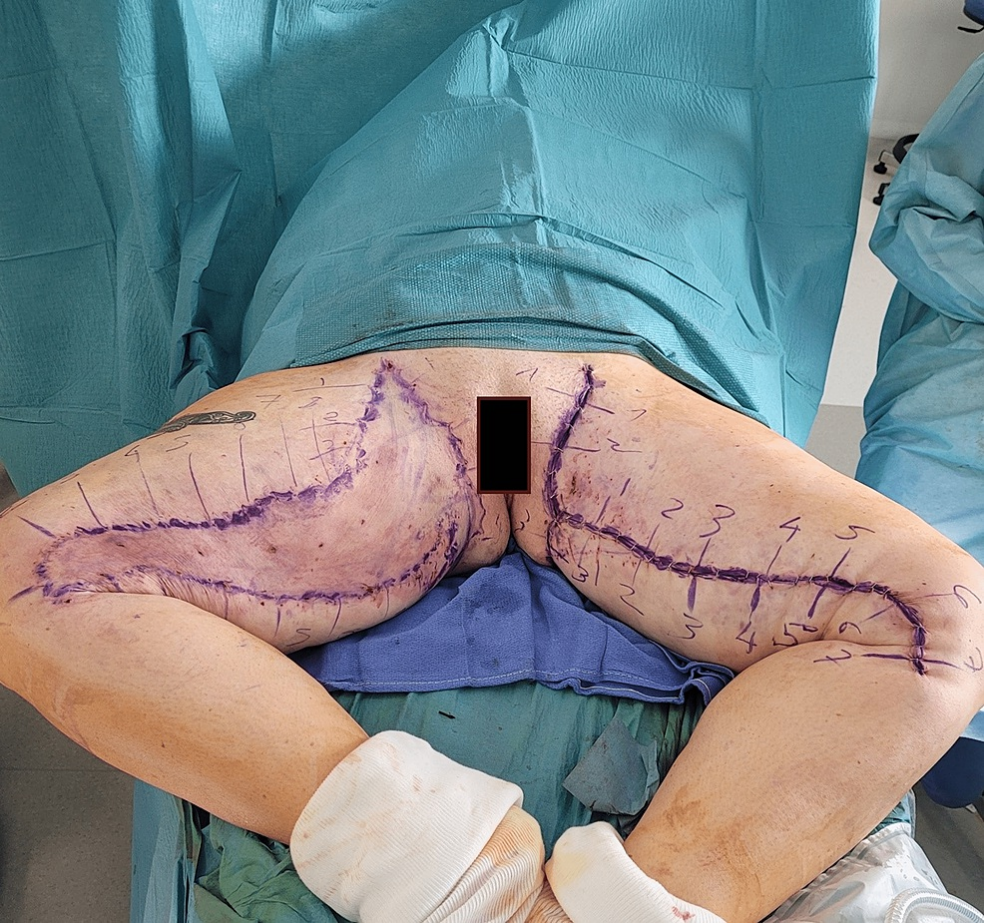
Staple frame and new markings The left side of the patient, staple frame with staples and new markings of resection area, and transverse markings. The right side of the patient, with new markings after removal of staples

Once the subcutaneous fat has been removed by liposuction inside the resection area, the excision drawings are checked using a preliminary staple frame. This maneuver allows for any necessary adjustments to the initial markings. Transverse markings are made to ensure proper alignment of the borders during closure (Figure 2).

**Figure 3:**
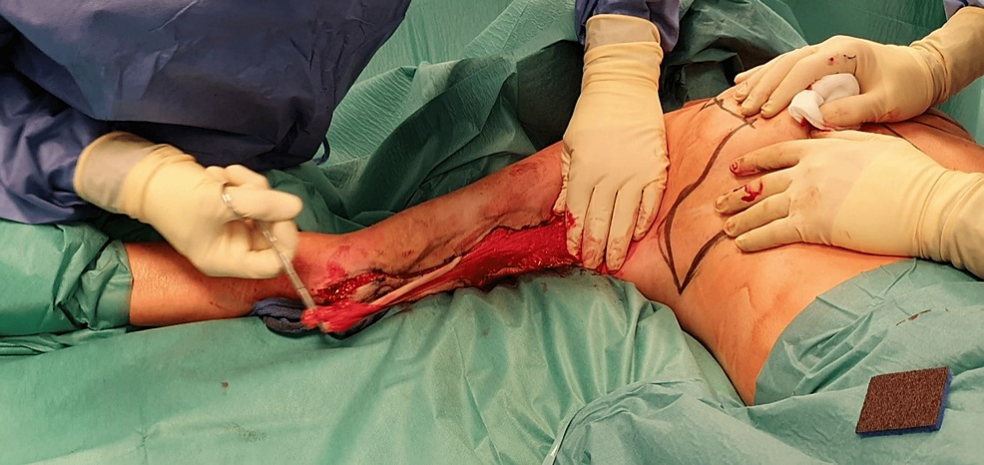
Skin avulsion with Kocher clamp

Once satisfactory markings are made, the staple frame is removed, and a skin incision is taken using a surgical blade. The operator then grasps the proximal end of the flap with a Kocher clamp. The skin is avulsed from proximal to distal while applying counterpressure with the other hand (Figure 3).

**Figure 4:**
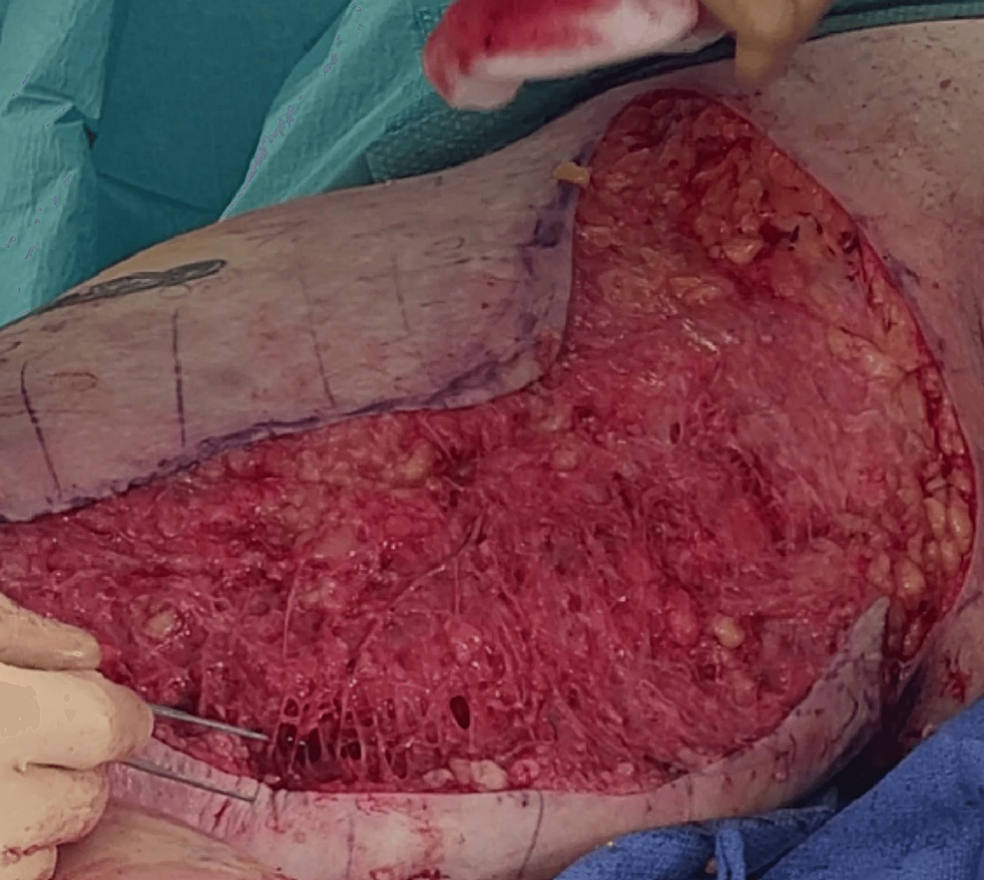
Immediate appearance after avulsion

Proximal-to-distal avulsion reduces the chances of avulsing vessels with the flap. Avulsion also allows for better preservation of blood vessels compared to excision with a cold blade or electrocautery. Almost no hemostasis is necessary after avulsion. Immediately after avulsion, we can see the preservation of superficial vessels and the absence of bleeding (Figure 4).

Closure

For cruroplasty, fixation to the Colles’ fascia is achieved using 2/0 polyglycolic acid sutures (Optime, Peters Surgical, Domalain, France). Five to six dermal support stitches are made using 3/0 polyglecaprone sutures (Monocryl, Ethicon Inc., Somerville, NJ) at the transverse marking sites, followed by a two-layer closure using 2/0 caprolactone sutures (Quill, Benewmedical, Melesse, France).

For brachioplasty, a few dermal support stitches are made using 3/0 polyglecaprone sutures (Monocryl, Ethicon Inc., Somerville, NJ) at the transverse marking sites, followed by a two-layer closure using 2/0 caprolactone sutures (Quill, Benewmedical, Melesse, France).

## Results

The avulsion technique systematically results in a thin flap with minimal subcutaneous tissue and conservation of the neurovascular framework.

A total of 11 patients who underwent brachioplasty and 17 patients who underwent cruroplasty were reviewed. The mean follow-up period was 22 months. Out of the 28 patients, 27 were females, with a mean age of 46 years and a mean preoperative body mass index (BMI) of 27. Among the 28 patients, 23 underwent combined surgeries involving additional procedures along with their surgery (nine brachioplasties and 14 cruroplasties). Complications assessed in the study included intrainterventional and postinterventional events related by Morzcycki within 6 months (Table 1).

**Table 1:** Intrainterventional and Postinterventional Adverse Events

Adverse Event	Number of Events
Intrainterventional	
Pain	0
Nerve injury	0
Tendon injury	0
Vascular injury	0
Laceration	0
Catheter trouble/failure of bolus	0
Greater than anticipated blood loss	0
Greater than anticipated blood loss requiring transfusion	0
Had to convert intraoperatively to alternative surgery type	0
Anaphylaxis	0
Syncopal event	0
Pneumothorax	0
Respiratory depression	0
Significant tachycardia	0
Unspecified	0
Postinterventional	
Infection	4
Reoperation	1
Hematoma	0
Seroma	0
Swelling	0
Impaired function	0
Recurrence of same problem	0
Abnormal scarring	0
Necrosis	0
Loss of sensation	0
Wound dehiscence	11
Erythema	0
Bruising	0
Complex regional pain syndrome	0
Tendon irritation	0
Pruritus	0
Skin rupture	0
Nausea/vomiting	0
Carpal tunnel syndrome	0
Malunion	0
Delayed wound healing	0
Bleeding	0
Death	0
Dermatitis	0
Device malfunction	0
Graft loss	0
Neuritis	0
Muscle spasm	0

Referring to the Common Terminology Criteria for Adverse Events (CTCAE) of the US Department of Health and Human Services, only grade 1 and 2 events were reported (Table 2). There were no cases of lymphedema, seroma, hematoma, necrosis, scar migration, or thromboembolic complications. Eleven patients experienced wound dehiscence (three brachioplasties and eight cruroplasties). Only one patient from cruroplasty had a large wound dehiscence of four centimeters that required scar revision at six months. Wound dehiscence was the most frequent complication, with no early occurrences and most cases appearing around postoperative day 15 in areas of high tension.

**Table 2:** CTCAE Classification for surgical and medical procedures

Grade 1	Grade 2	Grade 3	Grade 4	Grade 5
Asymptomatic or mild symptoms; clinical or diagnostic observations only; intervention not indicated	Moderate; minimal, local, or noninvasive intervention indicated; limiting age-appropriate instrumental ADL	Severe or medically significant but not immediately life-threatening; hospitalization or prolongation of existing hospitalization indicated; limiting self-care ADL	Life-threatening consequences; urgent intervention indicated	Death

Four patients had postoperative infections (two brachioplasties and two cruroplasties). The infections occurred distantly from the surgery and were managed with oral antibiotics and local care (grade 1 of CTCAE). Overall, 15 patients had postoperative complications (five brachioplasties and 10 cruroplasties). The total complication rate was 55% (45% for brachioplasties and 59% for cruroplasties). All complications were minor (grade 1 or grade 2 of CTCAE). No patients required rehospitalization or emergency reoperation. No drain was placed. All patients underwent outpatient surgery, except for two patients who required conventional hospitalization for combined surgeries.

**Figure 5:**
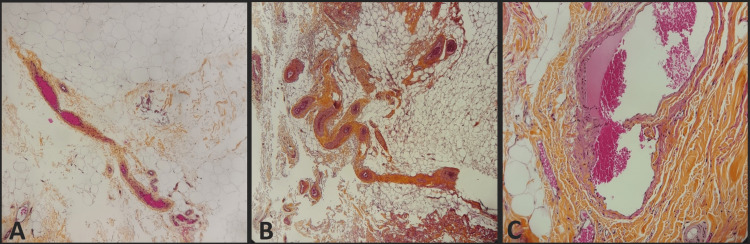
Histological analysis of skin and adipose tissue after a cruroplasty A: Normal aspect of vessels with electrocautery; B: Corkscrew aspect of vessels with avulsion; C: Intimal tear of hypodermic vessels

Histological analysis of avulsed tissues has revealed intimal tears responsible for thrombosis. Additionally, a corkscrew appearance has been observed, indicating significant tension exerted on the vessels during avulsion, which likely leads to the aforementioned lesions. These typical lesions were present in every sample obtained with avulsion, and any of them in electrocautery samples (Figure 5). The thickness of hypodermic tissue was macroscopically greater in the electrocautery group.

## Discussion

Despite advancements in brachioplasty and cruroplasty techniques, the main major complication that can occur during the postoperative period is the development of seroma, even with the use of liposuction. The occurrence of these complications is partly related to accidental injuries during the excision of the skin flap. Lymphatic injuries are common [15] and can be increased by using electrocautery and its heat radiation [5]. Vascular injuries, such as accidental damage to the great saphenous vein during cruroplasty, are also frequent. As for brachioplasties, which less frequently lead to lymphocele, accidental nerve injuries causing hypoesthesia remain rare but highly bothersome for patients.

Gusenoff et al. [15] reported a total complication rate of 74% in their study, with a seroma rate of 25% and a wound dehiscence rate of 51% for cruroplasties. Capella et al. [14] conducted a study involving 335 cases of cruroplasty and found a seroma rate of 19% despite the use of liposuction, with a wound dehiscence rate of 28.4%. However, the overall complication rate was not specified. Schmidt et al. [7] in a study involving 59 cruroplasties, demonstrated that aggressive liposuction statistically decreases the risk of overall complications (13% vs 59% (p<0.001)) and seroma (0% vs 33% (p<0.001)). Our results are consistent with those of Knotts and Hunstad who initially described the avulsion technique, as they reported 59% total complications, 49% wound dehiscence, 2% seroma, and 2% hematoma [8]. The complication rates associated with avulsion are similar to those reported in the literature.

They confirm that regardless of the technique used, aggressive liposuction is necessary to preserve the lymphatics that run superficially to the superficial facial system [5].The decrease in serious complications requiring urgent intervention, such as seromas and hematomas, combined with the absence of drainage, allows for outpatient management in the majority of cases. Lastly, the avulsion technique is a secure method that avoids rare but potentially severe accidental injuries (e.g., nerves, saphenous vein).

**Figure 6:**
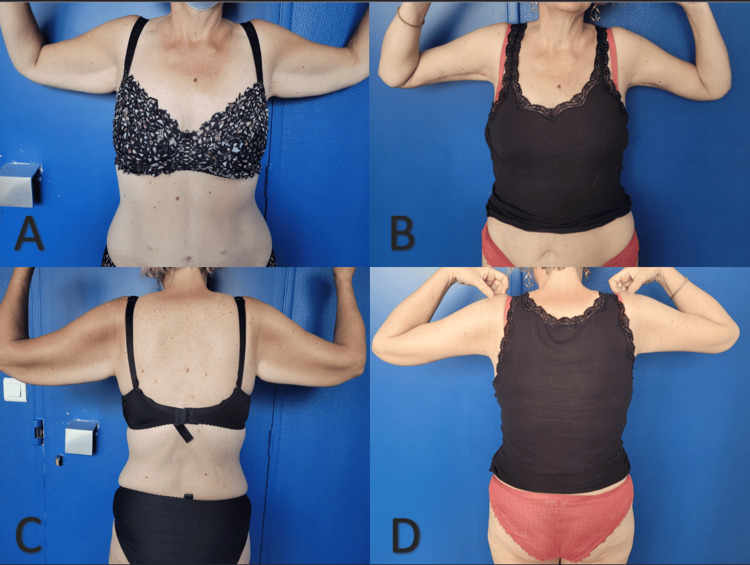
Result at three months of a bilateral brachioplasty A and C: Preoperative aspect; B and D: Appearance after 3 months

Extremity contouring surgeries are procedures with a low risk of major complications but a high risk of minor complications (55% in our study). Bertheuil et al. [16] were the first to confirm the improvement in quality of life provided by these interventions, which explains why patients are motivated to undergo these procedures despite the significant risk of scarring (Figure 6). During the preoperative consultation, it’s important for the surgeon to discuss these potential complications with the patient, as they are often well-accepted by the patients. Scar revisions are rare but can still be performed after complete healing. The evolutions of procedures reduce morbidity and allow for outpatient basis, which contributes to greater patient satisfaction. Conducting a prospective study with a large cohort of patients would help demonstrate the superiority of the modified avulsion technique in terms of complications such as seromas and hematomas, as well as operative time. Such a study would provide more robust evidence and further support the use of this technique in extremity contouring surgeries.

## Conclusions

Brachioplasties and cruroplasties are known to have a high rate of patient satisfaction and a low risk of major complications. Although minor complications such as small wound dehiscence are frequently observed, they do not significantly impact overall patient satisfaction. The avulsion technique used in extremity contouring procedures is considered safe and highly reproducible and has a short learning curve. In addition to saving time during excision and hemostasis, it ensures systematic preservation of the subcutaneous vascular tissues. The reduction of postoperative seroma eliminates the need for drain placement, allowing for ambulatory hospitalization and significant cost savings.
